# #ItsNotInYourHead: A Social Media Campaign to Disseminate Information on Provoked Vestibulodynia

**DOI:** 10.1007/s10508-020-01731-w

**Published:** 2020-06-02

**Authors:** Lori A. Brotto, Melissa Nelson, Lana Barry, Ciana Maher

**Affiliations:** 1 Department of Obstetrics and Gynaecology, University of British Columbia, 2775 Laurel Street, Vancouver, BC V5Z 1M9 Canada; 2 Women’s Health Research Institute, Vancouver, BC Canada; 3 Self-Management Programs, University of Victoria, Victoria, BC Canada

**Keywords:** Provoked Vestibulodynia, Knowledge translation, Psychosocial treatment, Pain management, CBT, Mindfulness, Social media

## Abstract

Provoked Vestibulodynia (PVD) is a type of localized vulvodynia (or pain in the vulva). The estimated prevalence of this condition is about 12% of the general population and approximately 20% of women under the age of 19. Many women who live with PVD suffer in silence for years before receiving a diagnosis. Whereas cognitive behavioral therapy (CBT) was already known to be effective for managing symptoms of PVD, there has recently been a published head-to-head comparison of CBT versus mindfulness-based therapy for the primary outcome of pain intensity with penetration. The trial revealed that both treatments were effective and led to statistically and clinically meaningful improvements in sexual function, quality of life, and reduced genital pain, with improvements retained at both 6- and 12-month follow-ups. We then undertook an end-of-grant knowledge translation (KT) campaign focused on the use of social media to disseminate an infographic video depicting the findings. Social media was strategically chosen as the primary mode of dissemination for the video as it has broad reach of audience, the public can access information on social media for free, and it presented an opportunity to provide social support to the population of women with PVD who are characterized as suffering in silence by starting a sensitive and empowering dialogue on a public platform. In this paper, we summarize the social media reach of our campaign, describe how and why we partnered with social media influencers, and share lessons learned that might steer future KT efforts in this field.

## Introduction

Provoked Vestibulodynia (PVD), characterized by provoked pain with touch to the vulvar and/or vaginal area, without obvious signs, affects up to 12% of women (Harlow et al., 2014; Pukall & Cahill, 2014; Reed et al., 2012) and is associated with a wide range of psychological, relational, and sexual difficulties (Basson, 2012; Desrochers, Bergeron, Landry, & Jodoin, 2008). Among sexually active young women under the age of 20, the prevalence of pain with intercourse for at least 6 months or more was found to be 20% (Landry & Bergeron, 2009). The economic burden of vulvodynia is $31–72 billion per year in the U.S. (Xie et al., 2012) including both direct costs (e.g., women with PVD often visit multiple healthcare providers and 40% of women with vulvar pain remain undiagnosed after seeking treatment) (Harlow & Stewart, 2003) and indirect costs (e.g., income loss due to time away from work). A more recent study estimated that the annual total direct costs, per patient with PVD, to the American healthcare system was over $117,000 per year, when pharmacy costs were included (Lua, Hollette, Parm, Allenback, & Dandolu, 2017).

Unfortunately, because of insufficient training to primary care doctors and gynecologists, many women are told the pain is “all in your head” when visible signs of tissue damage are not evident on exam (Brotto, Basson, Carlson, & Zhu, 2013; Kaler, 2006; Marriott & Thompson, 2008; Sadownik, Seal, & Brotto, 2012; Shallcross, Dickson, Nunns, Mackenzie, & Kiemle, 2018; Shallcross, Dickson, Nunns, Taylor, & Kiemle, 2019). Such messages further compound women’s sense of suffering and pain and elicited feelings of anger toward their healthcare providers (Shallcross et al., 2019).

### Evidence for Psychological Treatments of Provoked Vestibulodynia

There is growing evidence for psychosocial-based approaches to pain management. Among these approaches, cognitive behavior therapy (CBT) is the most commonly studied psychological treatment for PVD. One randomized trial comparing CBT to surgery (vestibulectomy) and pelvic muscle biofeedback found all three treatments to be effective for reducing vulvar pain intensity (as measured by cotton swab by a gynecologist) 6 months after treatment, although the vestibulectomy group showed the greatest reduction in pain during the cotton swab test of allodynia with a 70% reduction in pain in the surgery group and 29% reduction in pain in the CBT group (Bergeron et al., 2001). At a 2.5-year follow-up, pain intensity during intercourse was equivalent in the CBT and vestibulectomy groups (Bergeron, Khalifé, Glazer, & Binik, 2008), and reductions in pain intensity and improvements in sexuality outcomes (e.g., a global sexuality score that includes desire, arousal, orgasm, frequency of sexual activities, and overall satisfaction) were maintained for all three treatment groups (Bergeron et al., 2008). A pilot randomized trial comparing CBT to pelvic floor physical therapy also found comparable improvements in both groups, with effects maintained at 6 months after treatment (Goldfinger, Pukall, Thibault-Gagnon, McLean, & Chamberlain, 2016). Specifically, 70% of those in the CBT group and 80% in the physical therapy group showed a moderately clinically important decrease in pain. Another randomized study comparing individually delivered CBT to supportive group therapy administered weekly over 10 weeks found CBT led to significantly greater reductions in non-sexual provoked pain and improvements in sexual functioning compared to the support group (Masheb, Kerns, Lozano, Minkin, & Richman, 2009). Specifically, 39% of the participants receiving CBT had at least a 33% reduction in their vulvar pain intensity. One randomized trial compared 10 sessions of group CBT to an application of a topical steroid (consisting of 1% hydrocortisone cream) for 13 weeks for the primary outcome of pain during intercourse (Bergeron, Khalifé, Dupuis, & McDuff, 2016). Whereas participants in both conditions improved, women in the CBT group reported greater reductions in pain with intercourse, greater improvements in sexual function at the 6 month post-treatment time point, greater improvements in pain catastrophizing, and greater treatment satisfaction, but similar pain self-efficacy as compared to women receiving the topical steroid (Bergeron et al., 2016). Specifically, 68.6% of those in the CBT group reported good improvement to complete relief of pain at 6 months following treatment. Taken together, CBT has been recommended with Level 2 evidence as an effective treatment for pain intensity as well as sexual function and other associated psychological symptoms for women with PVD (Goldstein et al., 2016).

Though CBT is considered a second generation skills-oriented approach aimed at changing and challenging thoughts, newer, third wave approaches focus on cultivating the skill of acceptance. Mindfulness, a meditative practice defined as “non-judgmental, present-moment awareness” (Bishop et al., 2004) aims to increase awareness of, for example, pain-related thoughts and physical sensations with equanimity and without the intention of controlling or changing them. Stemming from the early work of Kabat-Zinn in the mid-1970s (Kabat-Zinn, 1982, 1990; Kabat-Zinn, Lipworth, & Burney, 1985), this mindfulness-based approach has been adopted by the general chronic pain field as an effective treatment for a number of chronic pain conditions compared to a wait-list control, treatment as usual, or to psychoeducation (Hilton et al., 2017; Kerns, Sellinger, & Goodin, 2011). Mindfulness promotes a state of awareness in which thoughts are allowed to reside in consciousness without any emotional attachment or aversion to them. It has been described as “uncoupling” the physical sensation from the emotional and cognitive experience of pain (Kabat-Zinn, 1982). Paying attention to physical sensations is distinctly processed from an experience’s emotional qualities, with the former processed in the inferior parietal and primary somatosensory cortices and the latter processed in the perigenual anterior cingulate and anterior midcingulate cortices (Kulkarni et al., 2005).

Recently, CBT has been compared to an equal duration mindfulness-based cognitive therapy (MBCT) intervention in a head-to-head trial focused on women with PVD (Brotto et al., 2019). Treatment consisted of eight 2-h weekly group sessions led by professional facilitators (who were sexual medicine physicians, psychologists, and upper level trainees in clinical psychology) who had expertise in mindfulness-based interventions, CBT, and managing PVD. Duration of sessions, assessments, and educational information about PVD were the same in both arms. The primary endpoint focused on vulvar pain intensity using a numeric rating scale (Farrar, Young, LaMoreaux, Werth, & Poole, 2001) and vulvo-vaginal pain assessed with a vulvalgesiometer (Pukall, Binik, & Khalifé, 2004; Pukall, Young, Roberts, Sutton, & Smith, 2007) designed to administer a fixed amount of pressure to the vulva. Additionally, several secondary endpoints focused on sexual functioning, sex-related distress, and various psychological outcomes used in studies of chronic pain.

Both treatments led to similar significant improvements in ratings of provoked vulvar pain using the vulvalgesiometer; overall sexual function; pain catastrophizing; pain hypervigilance; and sex-related distress. Though the effect sizes for both MBCT and CBT were large for the outcome of self-reported pain with vaginal penetration, the effect was greater for MBCT compared to CBT, suggesting potentially different mechanisms underlying these two treatments (Brotto, Bergeron, Zdaniuk, & Basson, 2020). All effects were in the moderate-to-very strong clinically meaningful range when assessed both 2–4 weeks after treatment and at the 6-month and 12-month follow-up periods (Brotto et al., 2019, 2020).

### Disseminating the Evidence

There is a gap in current practice and existing evidence when it comes to treating women with PVD. National surveys show that topical steroids and oral antidepressants are the most commonly used treatments by primary care physicians, yet scientific evidence does not find these treatments to be significantly more effective than placebo (Brown, Bachmann, Wan, & Foster, 2018; Foster et al., 2010). On the other hand, there is strong empirical evidence for two psychological approaches to managing PVD (CBT and mindfulness meditation; Brotto et al., 2019; Dunkley & Brotto, 2016; Goldstein et al., 2016). There is a need for women and their care providers to be informed of these evidence-based treatments so that women may receive care that leads to clinically meaningful and lasting improvements in their symptoms. Because PVD is associated with significant increases in depression and anxiety (Khandker, Brady, Stewart, and Harlow, 2014), and because ongoing and chronic mental health symptoms and stress complicate the management of women’s genital pain (Bachmann, Brown, & Foster, 2014), addressing mental health has been identified as essential if healthcare providers are to effectively move the needle on treating PVD (Sadownik, 2014). Women, themselves, acknowledge that they want healthcare providers to discuss the role of psychological factors in perpetuating their PVD, and that this is distinct from receiving the message that the pain is “all in their heads” (Shallcross et al., 2019).

In direct response to this state of affairs, we launched this knowledge translation (KT) project designed to disseminate evidence-based information directly to women with PVD in a social media campaign entitled #ItsNotInYourHead. KT is designed to address two well-known gaps in the translational continuum, which the Canadian Institutes of Health Research refers to as the “Valleys of Death.” The first gap lies between basic science and clinical science, and the second gap between clinical science and clinical practice, and these gaps have contributed to the often-cited figure of 17 years before new scientific data are adopted into practice (Morris, Wooding, & Grant, 2011). Furthermore, 14% of clinical research never makes its way to impact practice (Balas & Boren, 2000). Importantly, such gaps directly impact the care that patients receive when seeking treatment, and over half of physicians report not having adequate information to guide their treatment decisions (Dawes & Sampson, 2003; Kiesler & Auerbach, 2006; McGlynn et al., 2003). KT, also known as dissemination, is a set of strategies designed to share scientific information with target audiences (Kirchner, Waltz, Powell, Smith, & Proctor, 2017) and is widely recognized by funding agencies as a critical aspect of research. Research shows that women with PVD frequently go online to learn about different treatment options and treatment centers given their view that their healthcare providers lack key information about PVD (Shallcross et al., 2019).

The primary goals of this project were: (1) to develop a social media dissemination strategy and campaign and (2) to document reach by capturing metrics associated with various forms of social media. The long-term goal was to facilitate the uptake of scientific evidence by women (and other key stakeholders) who can directly utilize the new knowledge about PVD. Our goal was to maximize reach of #ItsNotInYourHead using a variety of tactics/strategies in the hope that women living with chronic genital pain would have access to information that might lead to an earlier diagnosis of PVD and which could facilitate conversations with their healthcare providers about possible treatment options. Since this project focused on reach, the latter putative outcomes were not measured.

## Method

### Knowledge Translation Framework

**Fig. 1:**
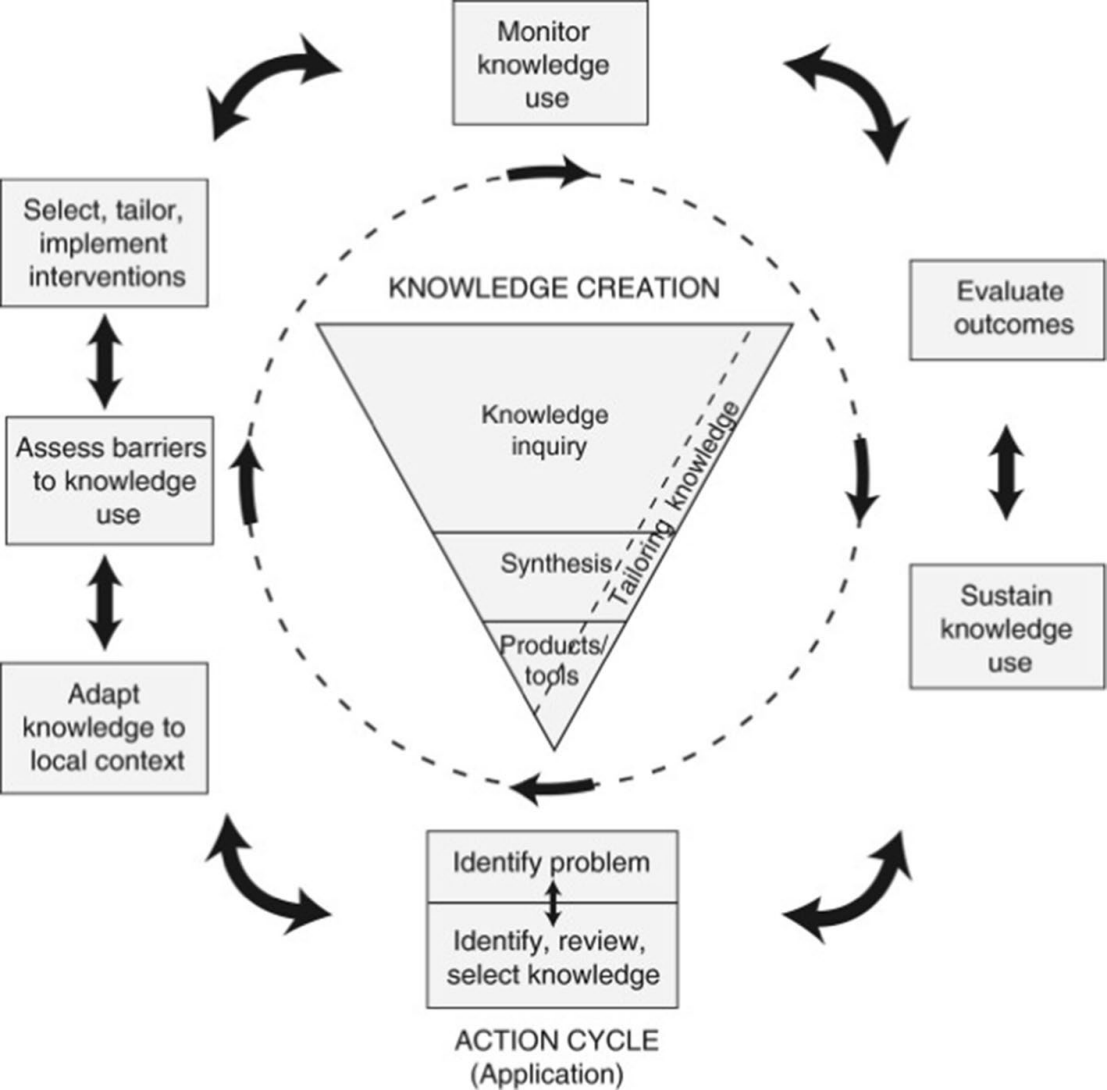
Knowledge-to-action (KTA) framework (Straus et al., 2009)

We were guided by the knowledge-to-action cycle framework (Straus, Tetroe, & Graham, 2009) which articulates the processes from knowledge creation to tailoring knowledge, to application of knowledge. There are two aspects of the cycle (Fig. 1): knowledge creation (represented by the middle funnel) and the action cycle (outer circle) which are seen as iterative and dynamic. This project focused on the centerpiece—knowledge creation, which includes knowledge inquiry (completion of primary research), synthesis (bringing different sources of research knowledge together), and production of tools. This project created knowledge toolkits and an infographic video. To facilitate our knowledge-to-action processes, we used the Knowledge Translation Planning Template (National Collaborating Centre for Methods and Tools, 2012) to develop our KT strategy. The checklist allowed our team to consider all stages of our knowledge translation strategy. We elected social media as our primary method of knowledge sharing given its exponential growth for communicating health-related topics to broad audiences (Hamm et al., 2013; Perrin, 2015). The template includes the following topics that should be considered in all KT projects: project partners (who are the partners on the team), degree of partner engagement (which aspect of the project will each partner participate in), partner roles (what does each partner bring to the project), KT expertise on the team (who holds which type of KT expertise on the team), knowledge users (which knowledge users or audiences will the KT activities target?), main messages (what messages are intended for the primary audiences?), KT goals (these should be specific to each knowledge user and audience), KT strategies (these should be informed by evidence of their effectiveness), KT process (will activities be integrated during the research or at the end?), impact and evaluation (where do you want to have an impact and how will that be evaluated?), resources (what outside supports are necessary for the KT activities), budget, and implementation (how will you implement the KT strategy). We chose the components that were most relevant to our current project and further elaborate on them below.

### Project Partners: Roles and Expertise

The core team consisted of a clinician-scientist with expertise in PVD and training in KT; a knowledge user partner who was a patient and research participant in studies of PVD; a communications assistant who had expertise in social media analysis and metrics; and a digital health research manager, with expertise in KT and digital health technologies. Additional team members include a patient advisory group, who developed the infographic video used in dissemination; a media design company (The Thinking Box); an award-winning digital marketing agency (Ehm&Co) designed to further boost the profile of the campaign; and five social media influencers who had audiences that aligned with our campaign values and who were contracted to amplify our campaign messages. The core team remained involved from initial project design through analysis, whereas the other team members participated at key time points throughout the campaign.

### Campaign Main Messages

The main messages we intended to disseminate through the campaign were: (1) chronic vulvar pain is common and you are not alone, and (2) there is evidence that psychological treatments can be effective in managing symptoms. The main KT goals were to generate awareness and interest in PVD and to impart knowledge. The target audience was women who may be experiencing chronic genital pain, regardless of whether they have been diagnosed with PVD or not. Our secondary audience members were healthcare practitioners, policymakers, partners, researchers, the media, and the general public. Because women’s decisions about healthcare may be directly influenced by their partners’ attitudes, by suggestions made by their healthcare providers, and by views of the general public, we believed it was important to also target these audiences in our dissemination activities.

### Strategies Employed

Social media was chosen as the primary KT strategy based on our prediction that our reach would be greatly enhanced compared to using other face-to-face or written vehicles for translation of this knowledge. Social media is the set of tools and networking platforms that allow people to connect, communicate, and collaborate using web-based technology (Jue, Marr, & Kassotakis, 2009). The advantages of social media to disseminate scientific information are well-documented (Hemsley & Mason, 2013; Oakley & Spallek, 2012) and include the rapid dissemination of information, the broad reach of the audience, the ability to create a community around a topic of interest, the use of metrics for evaluation of knowledge dissemination, flexibility in how to deliver information, the much faster exchange of information than face-to-face methods, and the ability to provide social support, which is of pertinence to this population of women with PVD who are characterized as suffering in silence. Moreover, Canadians find it acceptable to receive health information via social media (Royal College of Physicians and Surgeons of Canada, 2014) and there is evidence that Twitter is widely used and acceptable as a vehicle for increasing knowledge and exchanging advice (Antheunis, Tates, & Nieboer, 2013). In terms of KT process, we elected to follow an end-of-grant KT framework given that the findings from Brotto et al. (2019) were the basis of our social media messages. We set up a Twitter account, a public Facebook Page, a private Facebook Group, and an Instagram account which would be used to disseminate the video, key messages, and other related content during the campaign period. The accounts were all branded with the same colors and images from the infographic video to solidify the #ItsNotInYourHead brand.

Table 1 lists other strategies we used to increase engagement with our social channels and the content we promoted. Creation of the video took 6 months, followed by 2 months of meetings with the entire team prior to campaign launch. The campaign ran for 6 months from October 2017 to March 2018. Following this, 2 months were spent accumulating metrics from all sources, and a plain-language report was developed a month later.

**Table 1:** Strategies used throughout the #ItsNotInYourHead campaign

1.	Created original content to promote the campaign messages using the script, GIF clips, and stills from the #ItsNotInYourHead video
2.	Shared online media which featured Professor Lori Brotto discussing PVD and Mindfulness to promote the science supporting the campaign messages
3.	Consulted a patient partner with lived experience of the condition on the campaign team who helped promote content and gave a credible voice to the campaign
4.	Published 2–3 original tweets per week, 1 original Facebook post, and 1 Instagram post per week using the content management platform Hootsuite. We used images or graphics where possible to grab visual attention and boost post performance and used Hootsuite to monitor our hashtag, keywords, and several key accounts so we could join in and amplify online dialogues related to our campaign messages
5.	Tapped into existing online communities that dealt with chronic pain, women’s health issues, reproductive health issues, positive sex and leveraged the support of women’s health influencers and relevant organizations with an established following of our target audiences
6.	Hosted chats on our Twitter account with various groups to demystify some of the commons myths around PVD and shared evidence-based information regarding treatment of PVD
7.	Wrote blog posts promoting the campaign and trial findings for various outlets we knew had a following of our target audiences
8.	Aligned promotion with trending and viral hashtags, awareness days, or ‘take action weeks’ (e.g., #FactFriday, #MindfulnessMondays, World Compassion Day, Sexual Health Week, International Women’s Day, and National Pain Week)
9.	Developed an easily downloadable and user friendly social media toolkit which included template posts, graphics, and guidelines on how and when to use them on social media platforms
10.	Retrieved weekly social metrics to analyze what content was performing well so we could strategically target future posts (for example, specific content that received high engagement, days and times of day with most engagements)

### Infographic Video

The main dissemination product was a 143-s infographic video we created with a patient advisory group, our knowledge user partner, and with the company, The Thinking Box. The video (#ItsNotInYourHead) depicts a woman suffering in silence with chronic genital pain until she is diagnosed with PVD and suddenly realizes that she is not alone. The video then summarizes the results of our study (Brotto et al., 2019), and the video also depicts effective pain management techniques using both mindfulness-based and cognitive behavioral therapy based skills, highlighting that both of these treatments are effective pain management techniques for PVD. The final frame of the video lists useful resources where women might want to learn more about PVD.

As patient partners were fully engaged in the development of the video, they also named the campaign, #ItsNotInYourHead, emphasizing that the essence of the experience that women often face on their journey with chronic genital pain is frustration, sadness, and helplessness. In the creation of our infographic video, we were mindful of speaking to the diversity of individuals who may experience PVD and this was reflected in the illustrations.

### Social Accounts

Our primary outcome in this project was reach indicators, which can be defined as how many users are served campaign messaging on a given social platform or channel, and includes the accessibility of our video via social media based on our dissemination efforts. We used the following social accounts for dissemination: (1) Web: A webpage dedicated to the campaign was housed at www.whri.org. The page described how to use the campaign social media toolkit for dissemination and provided links to the video as well as our other social channels. (2) YouTube: The video was hosted on the Women’s Health Research Institute YouTube channel. (3) Instagram: We created the account @PVD_Advocacy to share our campaign. (4) Facebook: We created a public facing page @PVDadvocacy to promote our video and share information. We also created a private Facebook Group where women with PVD built a community of support. (5) Twitter: The handle @PVD_Advocacy was created to connect with women who experience symptoms of PVD.

In addition to targeted dissemination via the #ItsNotInYourHead social media channels, we collaborated with an award-winning digital marketing agency Ehm&Co to further boost the profile of the campaign and its key messages to the Yummy Mummy Club (YMC) community. Ehm&Co is the company behind yummymummyclub.ca. The partnership capitalized on the community’s monthly reach of over 5 million people. They created an integrated program for the #ItsNotInYourHead Campaign from January 2018 to March 2018 which included (1) a custom article shared through the YMC monthly newsletter and social media channels; (2) promotional posts about PVD and the campaign on YMC social channels; (3) a Twitter party (a sponsored live chat using the Twitter platform and hashtag (#) search feature to connect participants to an ultra-fast paced conversation stream on a specific topic); (4) a Facebook Live event; and (5) a Social Influencer Program. Individuals attending the Twitter Party are incentivized to join through an opportunity to win a monetary prize.

### Impact and Evaluation

We tracked the success of/metrics for our posts across all platforms, which was found to generate an international reach. Reach was defined as the number of users reached; impressions was defined as the number of times a user is served a post. (In other words, reach could be 12 unique users, whereas impressions could be 24 if those 12 users each saw the same post twice.)

We focused also on engagement, defined as the total number of times a user interacted with a post, including replies, follows, likes, links, cards, hashtags, embedded media, username, profile photograph, or post expansion. We also measured impressions, as indexed by the number of people who may have seen our content, regardless of whether it was clicked. All data presented are based on the campaign period of 6 months.

### Budget

Funding for this project came from a Knowledge Translation REACH award from the Michael Smith Foundation for Health Research ($9000). Additionally, the services of Ehm&Co ($28,000) were covered from an Operating Grant by the Canadian Institutes of Health Research to Brotto.

## Results

**Fig. 2:**
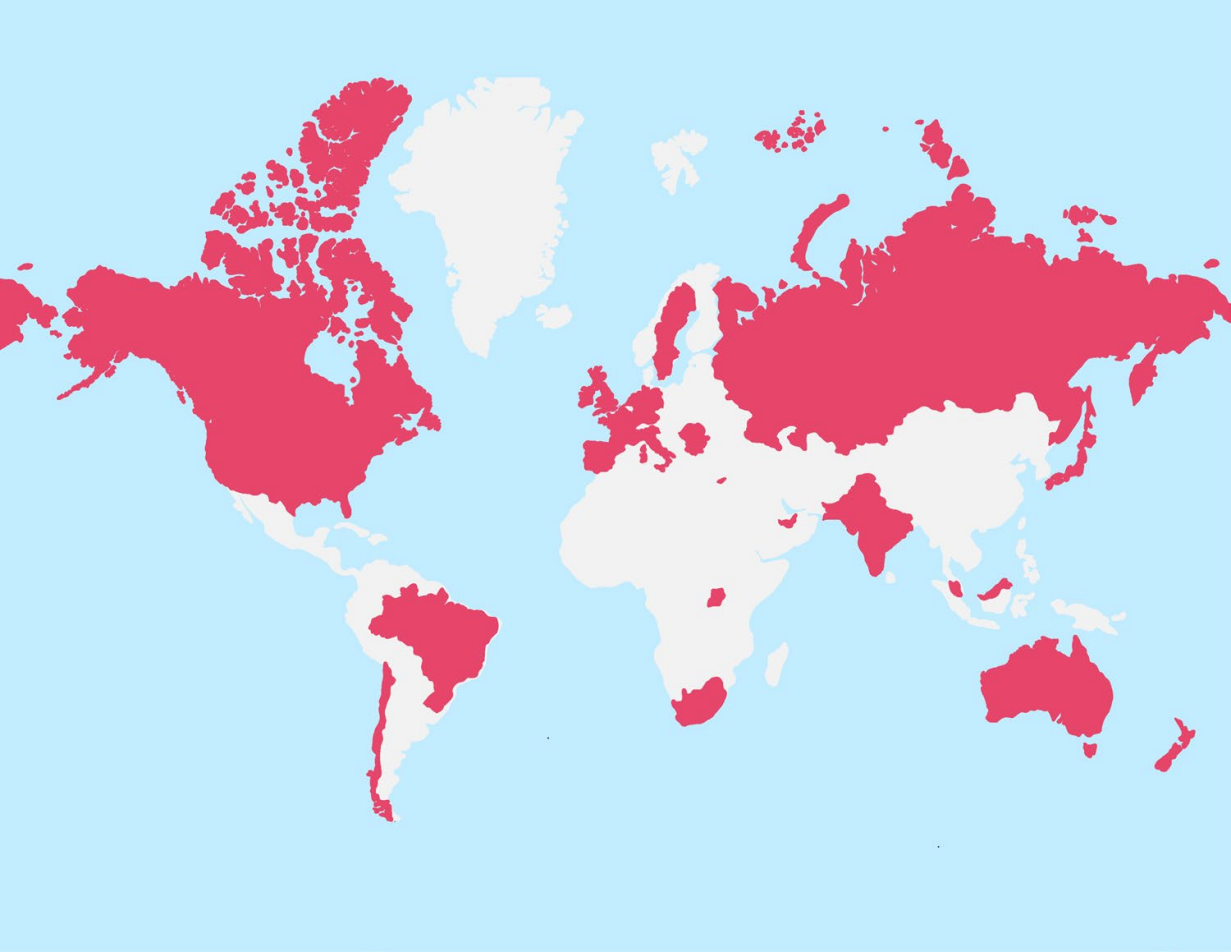
International reach of #ItsNotInYourHead Campaign. *Note* The colored areas indicate the reach of our campaign (Color figure online)

Over six months, our campaign reached a total of 45 countries (Fig. 2). Our webpage had a total of 180 unique page views, and our infographic video was viewed, on average, for 119 s (83% of video viewed). Direct views on YouTube were 785 for an average duration of 87 s (61% of video viewed) across 30 countries. All views of the video were in English, according to YouTube’s build-in analytics dashboard. Moreover, 11.4% of the total views added English subtitles. On Instagram we had 1077 Impressions, 253 Likes, and gained 40 followers. On Facebook, we had 53 followers and the highest reach on a single post was 198. On Twitter, we had 108,029 Impressions, 2307 Engagements, 402 retweets, 414 Likes, and 1047 media views.

**Fig. 3:**
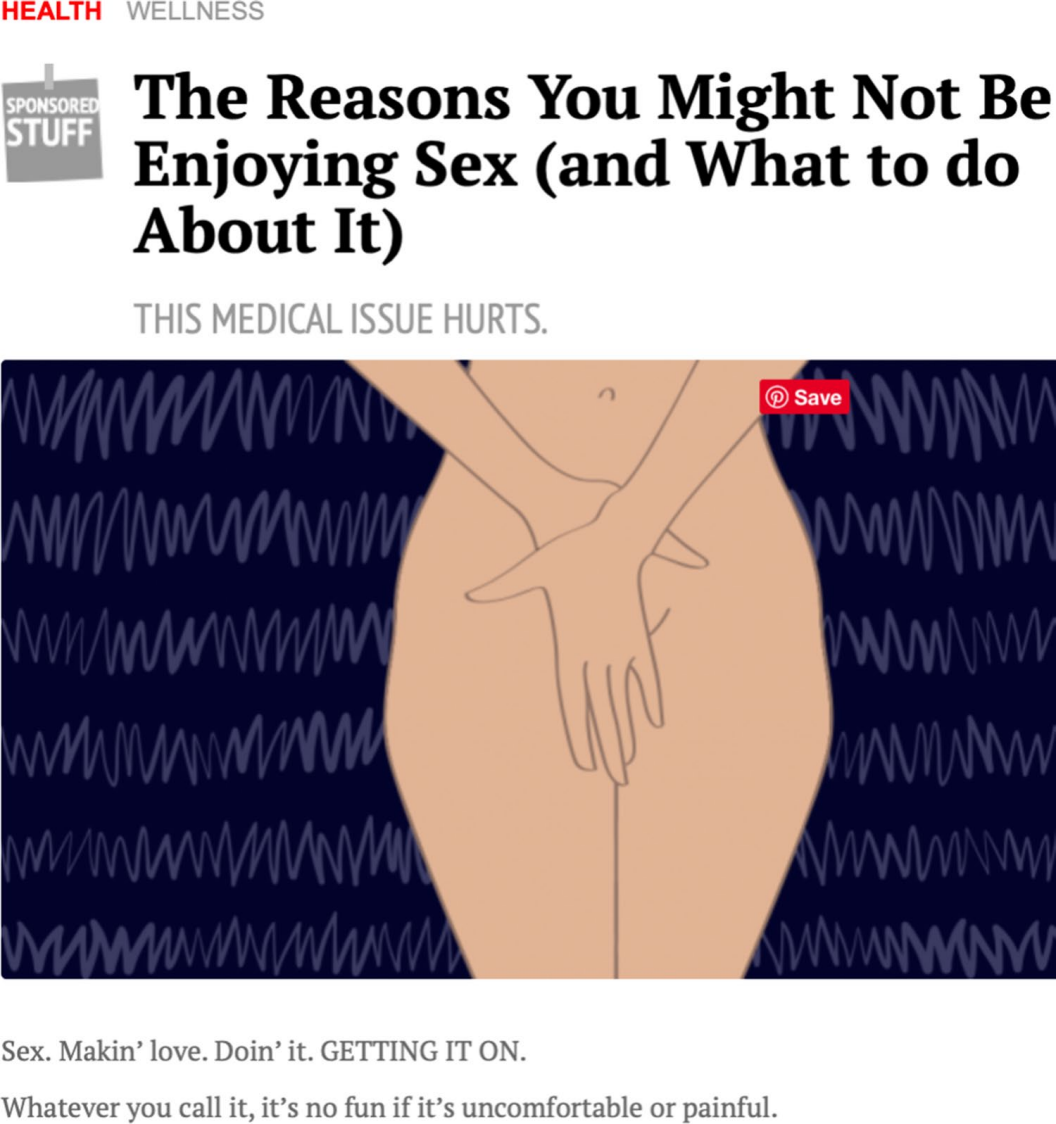
Yummy Mummy Club custom article

**Fig. 4:**
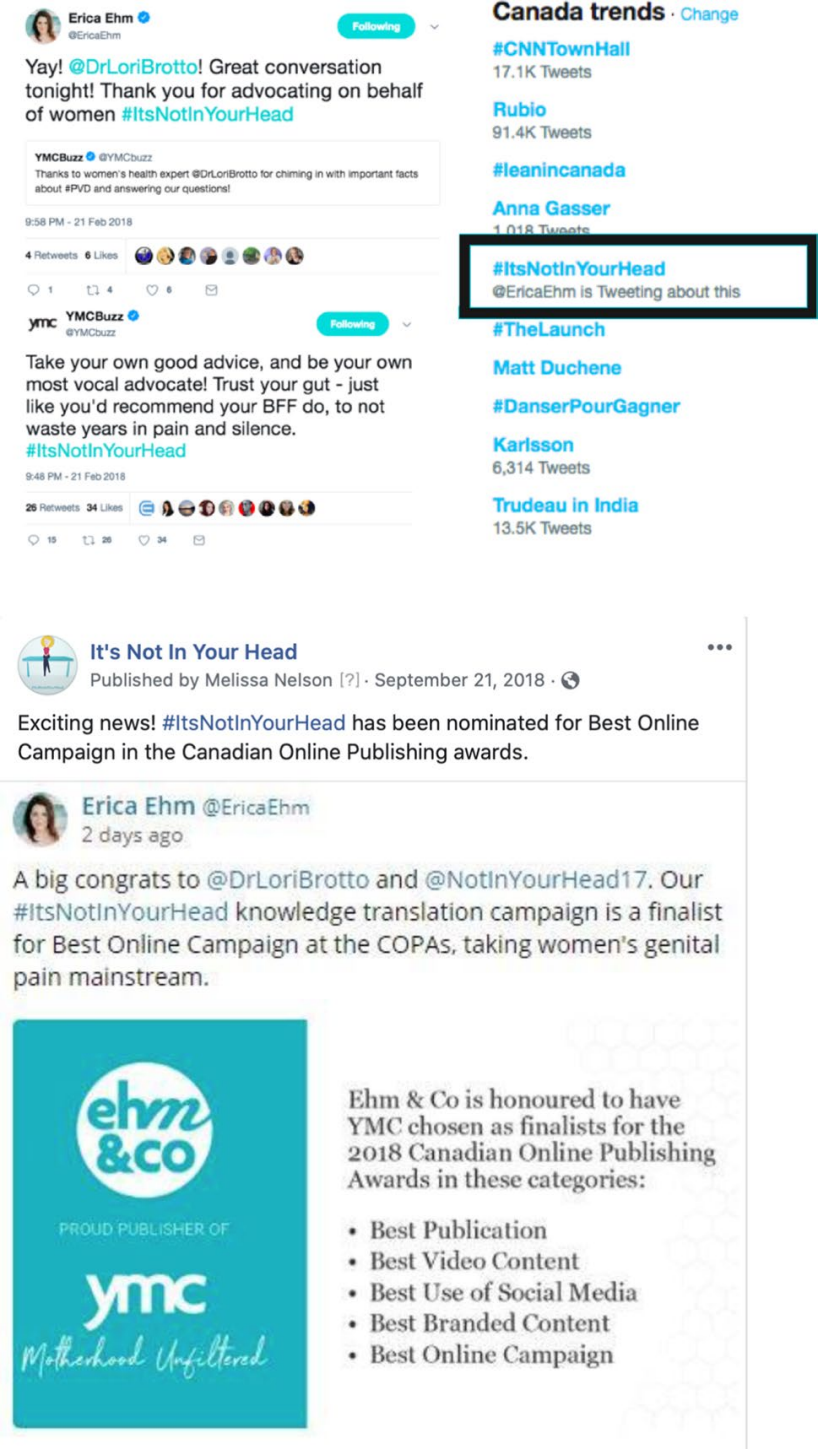
Online post showing the impact of our Twitter Party trending on Twitter Canada

Our campaign media partner, Ehm&Co, created a custom article about PVD and linked it to our campaign (Fig. 3) which had 1942 page views, 1161 Engagements, and an average viewing time of 204 s. Their social media posts (*n* = 29) generated 368,115 Impressions and led to 185 Engagements. They organized a Twitter Party consisting of a 1 h online chat with Brotto which generated 3400 tweets, 19,049,942 Impressions, 4873 Engagements, and included 101 participants. It also was on the top five trending in Canada (Fig. 4). Yummy Mummy Club also hosted a Facebook Live event during which Brotto answered questions live on video. This had a total of 30,900 views and 66 Engagements. Ehm&Co have a “social influencer program” which entailed engaging five influencers in the Yummy Mummy Club network who used key campaign messaging and imagery to share information about #ItsNotInYourHead and PVD with their audiences. A total of 30 posts were made leading to 1.5 million social Impressions and 3184 Engagements. In total, our partnership with Yummy Mummy Club led to 20.9 million Impressions.

Following completion of the campaign, #ItsNotInYourHead was named a finalist in the Canadian Online Publishing Awards for the category of best online campaign.

## Discussion

The goal of this project was to carry out a knowledge translation campaign designed to share information and raise awareness about Provoked Vestibulodynia to our primary target audience of women. By bringing together patients, clinicians, researchers, social media experts, digital design experts, and influencers, our campaign was designed to address a significant knowledge-to-action gap that has been well described by women with PVD (Shallcross et al., 2018, 2019). The team was guided by the knowledge-to-action (KTA) framework (Straus et al., 2009) and used the Knowledge Translation Planning Template (National Collaborating Centre for Methods and Tools, 2012) to develop our KT strategy. Throughout the 6-month campaign, our team’s communications assistant tracked metrics and reported these outcomes to the larger team at biweekly intervals.

Overall, we found that using all social outlets and all partners, our campaign reached 45 countries and led to over 21 million Impressions. As one measure of impact, we can conclude that our campaign reached its goal of sharing information about PVD. Moreover, the development of our infographic video, which was patient-partner led, was a key aspect of our KT plan as it combined evidence-based facts about PVD while also sharing the findings from a recent published clinical trial of psychological treatment for PVD (Brotto et al., 2019). We observed that viewers watched most of the infographic video (up to 83%), suggesting that this vehicle may be an effective way of translating scientific findings about PVD into an accessible format for women.

In 2 months following the end of the campaign, the team met to brainstorm, discuss, and then narrow down the factors that contributed to the success of our campaign suggests that three ingredients were key: having a dedicated campaign team, having a patient partner, and our media partnership with Ehm&Co. The campaign team consisted of researchers and clinicians with expertise in PVD, knowledge translation experts, a communications assistant who had expertise in social media analysis and metrics, a digital health research manager, and women with lived experience of PVD—all of whom were passionate about the #ItsNotInYourHead cause and message. Our patient partner was also a member of the investigator team since the project’s inception and this was seen as critical to the campaign’s success. Having the unique experiences of living with PVD, receiving treatment, as well as struggling to obtain evidence-based information about PVD through various means at pre-diagnosis meant we had the lens of our main target audience guiding us throughout the campaign as well as ensuring that the language we used throughout all of our posts was aligned with women’s experiences. Incorporating the patient voice throughout campaign activities added value in engaging women and disseminating the information in an accessible and relatable way. Other researchers investigating the experiences of women with PVD also advocate for patient engagement throughout the research development process (Shallcross et al., 2019), and we would advocate that this practice be standard among research studies designed to capture and reflect the lived experiences of women, particularly those with PVD. This has been described as a “paradigm shift” in health research where it has been concluded that “evidence-based medicine” is simply not possible without patient engagement (Sacristán, 2013).

Partnering with a digital marketing agency meant the campaign was amplified in a much more rapid manner and received extensive online exposure to a variety of audiences that may not have been reached through our own efforts. Ehm&Co’s editorial teams used their expertise in storytelling to translate the scientific findings in a way that resonated with a broad audience. This partnership also meant we added new online marketing tactics to our digital marketing tool box. Reflections shared by Ehm&Co suggested that this campaign, and this subject area, resonated with their community in a very special way, and they reported that their community wanted to learn more about PVD.

**Fig. 5:**
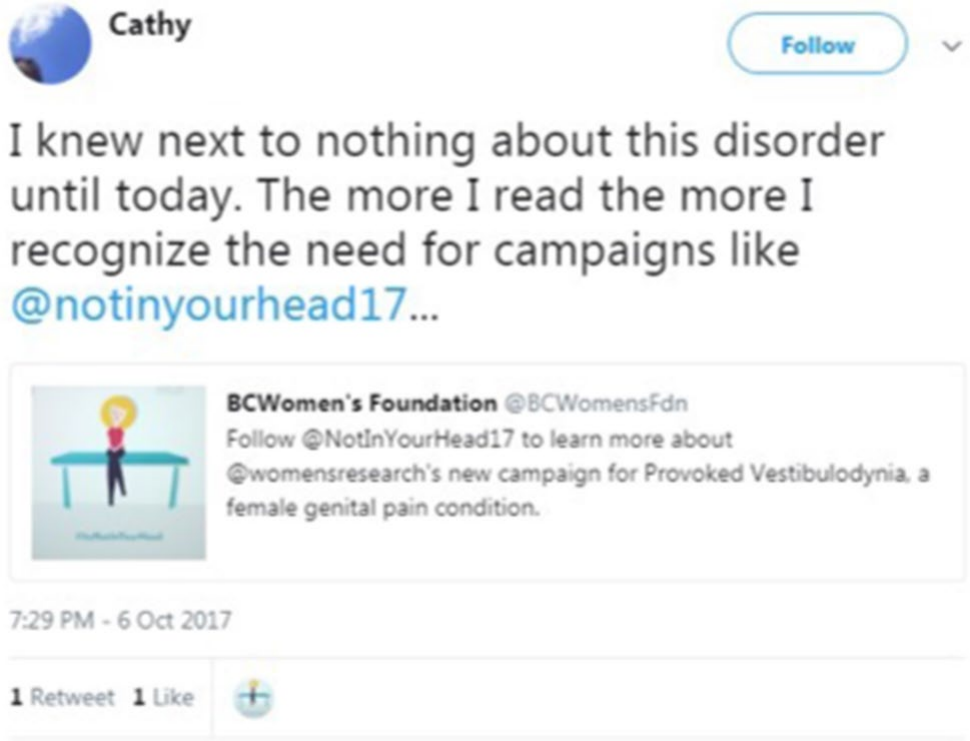
Sample tweet from a viewer of our social media campaign

Overall, we observed that among various social media strategies used, we generated the most reach and impressions with our Facebook Live event and particularly our Twitter Party, which trended on Twitter Canada (Fig. 4). Given that many women with PVD report suffering in silence, experiencing difficulty in obtaining evidence-based information about PVD, and being dissatisfied with their interactions with healthcare (Sadownik, 2014; Shallcross et al., 2018, 2019), the use of social media to share accurate information about PVD was seen to fill this gap. For many of our viewers, they expressed that this was the first time they had received evidence-based information about PVD (Fig. 5). Unfortunately, we did not assess women’s retention of this information, or if this led to any behavior change such as seeking a new healthcare provider, or making suggestions to their own healthcare providers about the availability of certain treatments that were highlighted during our campaign.

Knowledge translation emerged as a potential solution to bridge the known 17 year gap between science and practice (Morris et al., 2011). Since viewers watched most of our infographic video, it may be that healthcare providers can use this video as a means of providing women seeking their care with basic, standard information about PVD, and about the efficacy of psychological treatments. As a cost-effective means of sharing information about PVD, academic health center-approved social media accounts might be used to share evidence-based information from credible sources to women waiting to see healthcare providers. Furthermore, a professionally moderated social media account might be considered as a way of disseminating knowledge about PVD based on the empirical literature, and our findings suggest that the impact of its reach could be significant.

Furthermore, infographic videos might be a useful addition to the set of educational materials held by primary care doctors since they relay up-to-date and evidence-based educational information in a standard way to all patients. Our infographic video focused mostly on defining the symptoms of PVD and illustrating the process of obtaining a diagnosis. It then shifted to focus on the main outcomes of a large clinical trial. Women with PVD perceive their primary care doctors to lack basic information about PVD (Shallcross et al., 2019) and report this to be a barrier in their journey to wellness. Given that junior doctors, in particular, have been found to lack awareness and understanding of PVD due to lack of training (Toeima & Nieto, 2011), it may be that sharing the infographic video to their patients might offset some of this information gap. It is also the case that future KT efforts might specifically target (junior) primary care doctors who are likely the gate of entry into treatment for women with PVD.

Rurality is another factor that can directly impede access to health care (Humphreys, 2002), and there is evidence that women who live in rural and remote areas may not be receiving appropriate diagnostic and treatment information for PVD (Cox & Neville, 2012). Much is known about the predictors of sustainable e-health technologies in rural settings that help to bridge access to care issues (Hage, Roo, van Offenbeek, & Boonstra, 2013), and it may be the case that social media campaigns such as #ItsNotInYourHead may also be particularly useful for women with PVD living in rural and remote communities. Future studies should focus on the usefulness and reach of similar educational campaigns, specifically for women living in rural areas.

One limitation of our campaign is that although we could track geographic reach, we could not identify pertinent personal characteristics of those who engaged. For example, we do not know whether we reached our target audience of women, and how many of them had a diagnosis of PVD. We also could not measure whether viewers understood the information in our infographic video or whether the information shared was retained. Moreover, our campaign focused on raising awareness, but the potential impact on behavior remains unknown and particularly whether the information led to women who experience symptoms of PVD to receive a diagnosis more swiftly, or whether those with PVD were able to obtain evidence-based treatments more quickly. Behavior change theory posits that changing behavior is a complex process, whereby actual change in behavior may occur much later than informational and motivational changes (Prochaska & DiClemente, 1986). It remains a challenge for future social media campaigns to explore methods of extracting information about our viewers in order to assess whether strategies to reach the target audience were successful. Moreover, future projects need to incorporate methods of measuring behavior change after the target audience has received information. Finally, budget may be a barrier to other knowledge translation campaigns particularly if influencers must be compensated.

Guided by the Knowledge Translation Planning Template (National Collaborating Centre for Methods and Tools, 2012), we selected reach indicators as our metric of evaluating our KT goals. Other indicators of impact that might be used in a future KT campaign associated with sharing knowledge about PVD could include use indicators, practice change indicators, knowledge change, and attitude change. For example, a measure of use might be the number of PVD healthcare providers who have used the knowledge to make changes in their practice, including adding new educational information about PVD. A measure of practice change could be the number of units or clinics who intend to make changes as a result of the information learned. Knowledge change can be measured quantitatively and qualitatively and be assessed among women, healthcare providers, as well as the general public, for example. Attitude change might be captured by the number of women who no longer experience dismissive statements by healthcare providers.

Overall, we determined that this end-of-grant social media campaign designed to share information and raise awareness about PVD was successful. In addition to sharing general information about the diagnosis, it was used as an opportunity to share the findings from recent publications on psychological treatments for PVD (Brotto et al., 2019, 2020). Given the limited body of knowledge translation science in the field of women’s sexual health, this is a novel contribution to this body of literature, and we encourage the field to adopt this as a strategy for knowledge dissemination.
