# Correction to: #ItsNotInYourHead: A Social Media Campaign to Disseminate Information on Provoked Vestibulodynia

**DOI:** 10.1007/s10508-021-01970-5

**Published:** 2021-03-17

**Authors:** Lori A. Brotto, Melissa Nelson, Lana Barry, Ciana Maher

**Affiliations:** 1 Department of Obstetrics and Gynaecology, University of British Columbia, 2775 Laurel Street, Vancouver, BC V5Z 1M9 Canada; 2 Women’s Health Research Institute, Vancouver, BC Canada; 3 Self‑Management Programs, University of Victoria, Victoria, BC Canada

## Correction to: Archives of Sexual Behavior https://doi.org/10.1007/s10508-020-01731-w

The article “#ItsNotInYourHead: A Social Media Campaign to Disseminate Information on Provoked Vestibulodynia”, written by Lori A. Brotto, Melissa Nelson, Lana Barry, and Ciana Maher, was originally published electronically on the publisher’s internet portal on 2 June 2020 without open access. With the author(s)’ decision to opt for Open Choice the copyright of the article changed on 22 February 2021 to © The Author(s) 2021 and the article is forthwith distributed under a Creative Commons Attribution 4.0 International License, which permits use, sharing, adaptation, distribution and reproduction in any medium or format, as long as you give appropriate credit to the original author(s) and the source, provide a link to the Creative Commons license, and indicate if changes were made. The images or other third party material in this article are included in the article's Creative Commons license, unless indicated otherwise in a credit line to the material. If material is not included in the article's Creative Commons license and your intended use is not permitted by statutory regulation or exceeds the permitted use, you will need to obtain permission directly from the copyright holder. To view a copy of this license, visit http://creativecommons.org/licenses/by/4.0/.

